# Severe *Plasmodium vivax *malaria exhibits marked inflammatory imbalance

**DOI:** 10.1186/1475-2875-9-13

**Published:** 2010-01-13

**Authors:** Bruno B Andrade, Antonio Reis-Filho, Sebastião M Souza-Neto, Jorge Clarêncio, Luis MA Camargo, Aldina Barral, Manoel Barral-Netto

**Affiliations:** 1 Centro de Pesquisas Gonçalo Moniz (CPqGM), Fundação Oswaldo Cruz (FIOCRUZ), Salvador, Bahia, Brazil; 2 Faculdade de Medicina da Bahia, Universidade Federal da Bahia (UFBA), Salvador, Bahia, Brazil; 3 Departamento de Parasitologia, Instituto de Ciências Biológicas, Universidade de São Paulo, Brazil; 4 Faculdade de Medicina, Faculdade São Lucas, Rondônia, Brazil; 5 Instituto de Investigação em Imunologia (iii), Instituto Nacional de Ciência e Tecnologia (INCT), São Paulo, Brazil

## Abstract

**Background:**

Despite clinical descriptions of severe vivax malaria cases having been reported, data regarding immunological and inflammatory patterns are scarce. In this report, the inflammatory and immunological status of both mild and severe vivax malaria cases are compared in order to explore immunopathological events in this disease.

**Methods and Results:**

Active and passive malaria case detections were performed during 2007 in Buritis, Rondônia, in the Brazilian Amazon. A total of 219 participants enrolled the study. Study individuals were classified according to the presence of *Plasmodium vivax *infection within four groups: non-infected (n = 90), asymptomatic (n = 60), mild (n = 50) and severe vivax infection (n = 19). A diagnosis of malaria was made by microscopy and molecular assays. Since at present no clear criteria define severe vivax malaria, this study adapted the consensual criteria from falciparum malaria. Patients with severe *P. vivax *infection were younger, had lived for shorter time in the endemic area, and recalled having experienced less previous malaria episodes than individuals with no malaria infection and with mild or asymptomatic infection. Strong linear trends were identified regarding increasing plasma levels of C reactive protein (CRP), serum creatinine, bilirubins and the graduation of disease severity. Plasma levels of tumour necrosis factor (TNF), interferon-gamma(IFN-gamma) and also IFN-gamma/interleukin-10 ratios were increased and exhibited a linear trend with gradual augmentation of disease severity. Both laboratory parameters of organ dysfunction and inflammatory cytokines were reduced during anti-parasite therapy in those patients with severe disease.

**Conclusion:**

Different clinical presentations of vivax malaria infection present strong association with activation of pro-inflammatory responses and cytokine imbalance. These findings are of utmost importance to improve current knowledge about physiopathological concepts of this serious widespread disease.

## Background

*Plasmodium vivax *infection has been considered for a long time a benign and self-limited disease, mainly when compared to the burden of *Plasmodium falciparum *infection in African countries [1]. Nevertheless, *P. vivax *is responsible for up to 400 million infections each year, representing the most widespread *Plasmodium *species [2]. *Plasmodium vivax *accounts for the majority of malaria cases within the Brazilian Amazon [3], and the prevalence of asymptomatic infection is very high [4,5]. Historically, cases of complicated *P. vivax *malaria have been rare, and documented almost exclusively by case reports or small case series [6-8]. Recent evidence from larger studies performed in Melanesian populations has however reinforced the association between vivax malaria, severe complications, and death [9-11]. Severe complications associated with vivax malaria have also been reported in the Amazon region [12]. Together with rising documentation of drug resistance worldwide, the complications of *P. vivax *infection represent a global health menace which needs focused efforts to its resolution.

Major severe *P. vivax *clinical syndromes documented include important thrombocytopaenia [13,14], cerebral malaria [15,16], and acute renal [7,17], hepatic [6] and pulmonary [18,19] dysfunctions. In severe falciparum malaria syndromes, as in many other systemic infections, most of the pathology described seems to be a consequence of an intense inflammatory burst, favoured by a pathological activation of the immune system and cytokine release [20-22]. Despite clinical descriptions of the illness caused by *P. vivax *infection, data regarding immunological and inflammatory patterns are scarce. In the present report, inflammatory and immunological status of both mild and severe vivax malaria cases were compared in order to explore immunopathological events in this disease.

## Methods

### Study localities

A study investigating determinant factors for vivax malaria severity was performed during 2007 in Buritis (10°12'43" S; 63°49'44" W), a recent urbanized municipality of the Rondônia State, in the south-western part of Brazilian Amazon. Within this region, malaria transmission is unstable, with increased number of cases being detected annually between April to September, and the risk of infection is high [23], with an Annual Parasite Incidence of 77.5 per 1,000 inhabitants in 2005 [3]. The prevalence of *P. falciparum *infection in the Brazilian Amazon is 23.7% [3], and *Plasmodium malariae *case detection reaches 10% in Rondônia [24].

### Participants and sampling

Active and passive malaria case detections were performed. These included home visits in areas of high disease transmission, and study of individuals who seek care at the diagnostic centers of Brazilian National Foundation of Health (FUNASA), responsible for malaria control in the country. In addition, patients admitted to the Buritis municipal Hospital (Hospital São Gabriel) presenting clinical signs of mild or complicated malaria were also included in the study. All individuals from fifteen to seventy years, of both sexes, who had been residing in the endemic area for more than six months, were invited to be included in the study. Exclusion criteria were: documented or strong clinical suspecting of viral hepatitis (HAV, HBV, HCV, HDV), chronic alcoholism, HIV infection, yellow fever, dengue, leptospirosis, tuberculosis, Hansen's disease, visceral leishmaniasis, documented or referred cancer and/or other chronic degenerative disease, sickle cell trait, and the use of hepatotoxic and immunessupressive drugs. All participants or legal responsible gave written informed consent before entering the study. This study was approved by the Ethical Committee of the São Lucas University, Rondônia, Brazil, for the human subject protocol.

Individuals were examined and interviewed and their blood samples (20 mL) were collected for serological experiments. In hospitalized participants, two venous blood collections were performed: one at the hospital admission and other seven days after malaria treatment initiation. All malaria diagnoses were performed using two methods. First, patients were screened by thick smear examination using field microscopy and the parasitaemia (parasites/uL) was quantified in positive cases. Further, nested PCR was performed in all whole blood samples to confirm the diagnosis. Two individuals presenting *P. malariae *infection and 16 people infected with *P. falciparum *(uncomplicated forms) were identified and excluded from the study. Hence, all the volunteers selected were negative for *P. falciparum *and/or *P. malariae *infection by both microscopic examination and nested PCR.

A total of 219 individuals enrolled in the study. All positive cases were followed for 30 days for the evaluation of malaria symptoms. Individuals who were positive for *P. vivax *infection and remained without fever (axillary temperature >37.8°C) and/or chills, sweats, strong headaches, myalgia, nausea, vomiting, jaundice, asthenia, and arthralgia for 30 days were considered asymptomatic *P. vivax*-infected cases. Cases showing positive parasitological tests in the presence of any symptom listed above were classified as symptomatic infections. Patients presenting any sign of acute severe organ dysfunction [25] were considered severe cases. Until today there are no clear criteria defining what a severe vivax malaria case is. Despite the absence of a consensus, this study used the previously defined criteria for severe falciparum infection [25]. Study individuals were then classified within four groups: non-infected (n = 90), asymptomatic (n = 60), mild (n = 50) and severe vivax infection (n = 19). The baseline characteristics of the volunteers are listed in the Table 1.

**Table 1:** Baseline characteristics of the participants.

	*Plasmodium vivax *infection			
	
Variables	Non-infected N = 90	Asymptomatic N = 60	Mild N = 50	Severe N = 19
**Male - no. (%)**	39 (43.3)	30 (50.0)	22 (44.0)	10 (52.6)
Age - year *				
Median	38.0	42.0	33.0	22.0
Interquartile interval	25.0 - 51.0	32.0 - 48.2	26.7 - 48.0	16.0 - 35.0
Previous malaria episodes *				
Median	14.0	16.0	8.0	3.5
Interquartile interval	10.0 - 18.0	13.0 - 20.0	1.0 - 12	2.0 - 7.5
Years resident in the area *				
Median	11.4	12.5	7.4	3.0
Interquartile interval	3.2 - 12.8	4.2 - 14.6	0.5 - 9.2	0.5 - 5.4
Parasitaemia (parasites/uL) *				
Median	0	73 §	4,798	49,358
Interquartile interval	0	54.0 - 85.0	2,934 - 7,483	32,796 - 54,244
Haemoglobin (g/dL) *				
Median	13.2	11.5	8.9	6.4
Interquartile interval	9.2 - 14.5	9.5 - 14.2	7.3 - 12.6	5.8 - 7.4
CRP (ng/mL)*				
Median	5.65	6.6	6.5	15.3
Interquartile interval	3.7 - 9.47	4.12 - 9.35	4.9 - 8.7	11.9 - 20.65
Serum creatinine (mg/dL)*				
Median	0.85	0.9	1.1	1.7
Interquartile interval	0.7 - 1.2	0.7 - 1.2	0.7 - 1.3	1.42 - 2.45
AST (U/L)*				
Median	41.5	50.2	95.2	385.5
Interquartile interval	32.5 - 68.3	38.4 - 73.5	42.6 - 251.7	277.3 - 487.4
UNL	1.04	1.25	2.38	9.64
ALT (U/L)*				
Median	42.35	40	58.3	238.4
Interquartile interval	37.28 - 53.58	23.25 - 65.78	43.6 - 87.5	105.5 - 364.6
UNL	1.06	1	1.46	4.96
Total bilirubin (mg/dL)*				
Median	0.35	0.4	0.8	2.1
Interquartile interval	0.3 - 0.4	0.3 - 0.62	0.7 - 2.05	1.15 - 3.1
**Direct bilirubin (mg/dL)**				
Median	0	0.11	0.3	1.1
Interquartile interval	0 - 0	0.01 - 0.4	0 - 1.63	0 - 2.2
**Indirect bilirubin (mg/dL)**				
Median	0.3	0.28	0.5	1.1
Interquartile interval	0.28 - 0.37	0.2 - 0.3	0.45 - 0.72	0.6 - 1.3

CRP: C reactive protein; AST: aspartate aminotransferase; ALT: alanine amino-transferase; UNL: Upper normal levels. Data represent the number of times the median of AST or ALT is higher than the standardized normal laboratory level (40 U/L). Ordinal variables were compared using the Kruskal Wallis test with Dunn's multiple comparisons. The prevalence of male gender was compared between the groups using chi-square test. §Six out of sixty individuals with asymptomatic *P. vivax *infection were negative for malaria infection by light microscopy, but were positive for *Plasmodium vivax *infection by nested PCR. *Differences were significant between groups (P < 0.05).

### Nested PCR for malaria diagnosis

The molecular diagnosis of malaria infection was performed in all subjects using the nested PCR technique described previously [26,27], with minimal alterations [28]. To control for cross-contamination, one uninfected blood sample was included for every twelve samples processed. Fifteen percent of positive PCR samples were retested to confirm the amplification of plasmodial DNA. All tests were performed and confirmed at the Centro de Pesquisas Gonçalo Moniz, Salvador, Bahia, Brazil.

### Plasma cytokine levels detection

Plasma levels of IL-10, IFN-gamma, and TNF were measured using the Cytometric Bead Array - CBA (BD Biosciences Pharmingen, USA) according to the manufacturer's protocol, with all samples running in a single assay. The flow cytometric assay was performed and analysed by a single operator, and standard curves were derived from cytokine standards.

### Laboratory assessment of organ dysfunction

Plasma measurements of creatinin, aspartate amino-transferase (AST), alanine amino-transaminase (ALT), total and direct bilirubins, haemoglobin, and CRP were made at the clinical laboratory of Faculdade São Lucas, at the Pharmacy School (Federal University of Bahia, Brazil) and at the Laboratório LPC (Salvador, Bahia. Brazil).

### The Hepatic-Inflammatory Parasitic score

**Figure 1:**
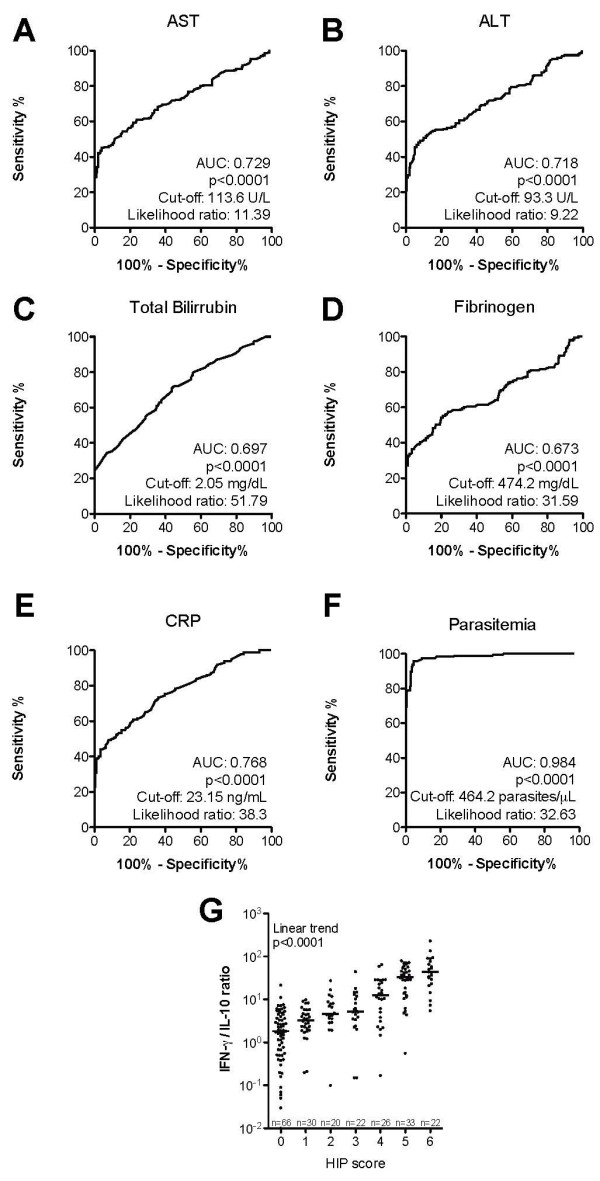
**The Hepatic-Inflammatory Parasitic score**. In a primary investigation, 580 individuals from Buritis, Rondonia, Brazil were evaluated to standardize the Hepatic-Inflammatory Parasitic (HIP) score. This sample included non-infected individuals (n = 183) and those infected with *Plasmodium *presenting malaria-related symptoms (n = 195) or asymptomatic infection (n = 202). The threshold plasma values of (A) aspartate aminotransferase (AST), (B) alanino amino-transaminase (ALT), (C) total bilirubin, (D) fibrinogen, (E) C reactive protein (CRP) and (F) parasitaemia were established in order to categorize the individuals according to the HIP score. Once the HIP score was created, it was applied in another sample from the same endemic area composed by 219 individuals: non-infected (n = 90), asymptomatic (n = 60), mild (n = 50) and severe vivax infection (n = 19). Area under the curve (AUC) was calculated, together with the cut-off value, which presents the higher likelihood ratio, and P values are plotted. The HIP score is described in Methods.

The hepatic-inflammatory parasitic (HIP) score was created to standardize a reproducible evaluation of severity in malaria cases. This score was developed by analysing data from another study conducted in 2006 with a sample size of 580 individuals from the Buritis Municipality, Rondônia State, Brazil. This group was composed of non-infected individuals (n = 183) and those infected with *Plasmodium *presenting malaria-related symptoms (n = 195) or asymptomatic infection (n = 202) composed this sample. In addition, this group was very similar to the one in 2007 with regard to age, gender, time of residence in endemic area and referred previous malaria episodes (data not shown). Optimal threshold plasma values of AST, ALT, total bilirubin, fibrinogen, CRP, and parasitaemia able to discriminate asymptomatic from symptomatic malaria infection were calculated using the Receiver Operator Characteristics (ROC) curves (Figure 1). For each variable measured, the cut-off values presenting the higher sensitivity and specificity, as well as the highest likelihood ratio, were established (Figure 1A-F). Further, one point was attributed to each variable that presented higher than the established cut-off value. Consequently, the minimum score was zero and maximum was five, and it reflected both parasitaemia and organ dysfunction aspects of symptomatic disease. Once the score was established, it was tested by applying to the sample constituted by the 219 participants approached in this study (Figure 1G). Additionally, the relationship between the HIP score and the IFN-gamma/IL-10 was assessed, since this ratio has been used as indicator of inflammatory activity in malaria [21,22,29].

### Statistical analysis

Data were analyzed using the GraphPad Prism 5.0 (GraphPad Software Inc.). For the ordinal variables, differences between groups were calculated using the non-parametric Kruskal-Wallis test with Dunn's multiple comparisons or trend analysis. The chi-square test was used to compare differences in categorized variables. The correlations were assessed using the Spearman test. Non-linear curve fit was also plotted to illustrate the general trend of the correlations. The statistical analyses used are illustrated in each figure or table. Differences presenting P ≤ 0.05 were considered statistically significant.

## Results and Discussion

### Baseline characteristics and laboratory assessment of *P. vivax *infection severity

The majority of the participants were male, with no gender differences among groups (P = 0.78). As previously described [4,28], individuals with asymptomatic *P. vivax *infection were older, had experienced more previous malaria episodes and presented lower parasitaemia than had symptomatic cases (Table 1). Patients with severe *P. vivax *infection were younger, having lived for a shorter time in the endemic area, and had experienced fewer previous malaria episodes than individuals with no malaria infection and with mild or asymptomatic infection (Table 1). Moreover, patients with severe disease displayed higher parasitaemias than those with uncomplicated infection (P < 0.0001). Haemoglobin levels were also decreased in patients with severe disease (P = 0.02). All patients with severe disease were admitted to the municipal hospital presenting with fever, tachycardia and tachypnea. Moreover, five out of nineteen individuals with severe infection developed jaundice and six presented with splenomegaly. Six infected patients died within 72 h of hospitalization, four presenting with acute respiratory failure and two with anuric renal failure, despite the haemodynamic support and anti-parasite therapy. These severe complications have been commonly implicated as major death causes in severe vivax infections [7,18]. The other thirteen individuals with complicated disease received specific treatment with intravenous quinine and achieved total clinical recovery after 10-15 days. Clinical characteristics and outcomes of the patients with severe malaria are summarized in Table 2. All patients with mild disease recovered totally and no drug resistance was identified within individuals studied.

**Table 2:** Characterization of the patients with severe vivax malaria.

			Clinical presentation at admission		*P. vivax *diagnosis		
				
**Patient No**.	Gender	Age (y)	Major manifestation	Secondary manifestation	Nested PCR	Microscopy	Outcome
1	M	15	Oliguria	Hypotension, splenomegaly	+	+	Recovered
2	M	17	Respiratory failure	Hypotension	+	+	Recovered
3	F	9	Respiratory failure	Hypotension	+	+	Died
4	M	21	Severe anaemia	Hypotension, splenomegaly	+	+	Recovered
5	M	22	Severe anaemia	Hypotension	+	+	Recovered
6	M	32	Anuric renal failure	Hypotension, splenomegaly	+	+	Died
7	F	41	Respiratory failure	Hypotension, Jaundice	+	+	Died
8	F	15	Severe anaemia	Splenomegaly	+	+	Recovered
9	M	15	Anuric renal failure	Hypotension, Jaundice	+	+	Died
10	M	17	Severe anaemia	Splenomegaly	+	+	Recovered
11	F	13	Jaundice	Splenomegaly	+	+	Recovered
12	F	26	Jaundice	Hypotension	+	+	Recovered
13	M	32	Respiratory failure	Hypotension	+	+	Recovered
14	F	27	Seizure	Jaundice	+	+	Recovered
15	M	42	Oliguria	Hypotension	+	+	Recovered
16	F	38	Jaundice	Hypotension	+	+	Recovered
17	F	54	Respiratory failure	Hypotension	+	+	Died
18	M	24	Severe anaemia	Hypotension	+	+	Recovered
19	M	22	Respiratory failure	Jaundice	+	+	Died

Data regarding major and secondary manifestations were obtained from medical records and/or through the clinical exam at the hospital admission. Oliguria was defined as estimated urinary output less than 400 mL/24 h and anuric renal failure as urinary output below 100 mL/24 h. Severe anaemia was defined as haemoglobin levels below 7 g/dL and jaundice by clinical exam and bilirubin levels above 2.0. Hypotension was defined as the presence of related symptoms with blood pressure below 100 × 40 mmHg. Respiratory failure was defined as tachypnea, shortness of breath, mental confusion clinical signs of hypoxaemia (central and/or peripheral cyanosis).

The further step was to assess whether the spectrum of vivax malaria clinical presentation could be associated with laboratory parameters of organ dysfunction. In a primary analysis, strong linear trends were identified regarding increasing plasma levels of CRP, serum creatinine, bilirubins and the graduation of disease severity (Table 2; P < 0.0001 for all trends analyzed). The individuals presenting higher HIP scores also displayed elevated IFN-gamma/IL-10 ratios (Figure 1G). These data indicate that a high grade of general inflammation-mediated systemic damage is occurring in some vivax malaria cases, explaining the severity of their clinical presentations.

### Inflammatory balance according to *P. vivax *infection severity

**Figure 2:**
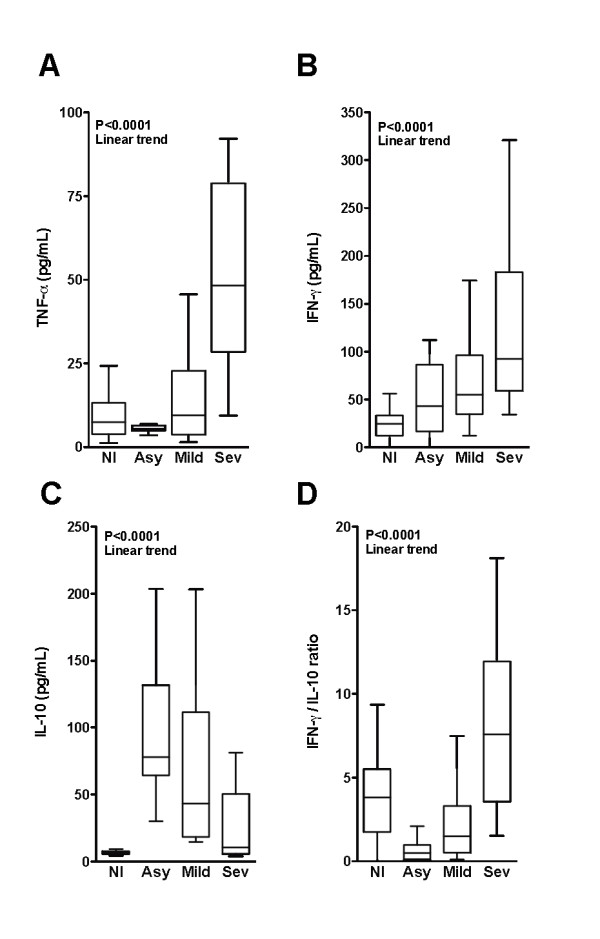
**General trend of the Inflammatory profile in vivax malaria**. Plasma levels of (A) TNF, (B) IFN-gamma, (C) IL-10 and (D) IFN-gamma/IL-10 ratios were estimated in non-infected individuals and those presenting different manifestations of the vivax malaria clinical spectrum. Study participants were stratified in groups as follows: non-infected (NI; n = 90); asymptomatic infection (Asy; n = 60); mild infection (Mild; n = 50); and severe infection (Sev; n = 19). One-Way ANOVA with trend analysis was performed to check the statistical significance between the groups studied. P values are plotted in each graph.

Furthermore, a possible link between the differences in clinical presentation and laboratory parameters of organ damage and specific patterns of immune responses or inflammatory mediators profile was evaluated. Plasma TNF, which is related to *P. vivax *paroxysms [30], was higher according to infection severity (Figure 2A). IFN-gamma is also implicated in both resistance to malaria [31] and disease immunopathology [32]. In the present series, IFN-gamma levels were higher in patients with increased severity (Figure 2B). Interestingly, the increasing levels of all these inflammatory markers also presented a linear trend with the gradual augmentation of infection severity (P < 0.0001 for each parameter). Conversely, plasma levels of IL-10, a cytokine that down-regulates inflammation, were lower with increased disease severity (P < 0.0001, for linear trend; Figure 2C). Thus, IFN-gamma/IL-10 ratio values were higher in patients with increased disease severity (P < 0.0001, for linear trend; Figure 2D).

### Kinetics of inflammatory responses during the treatment of severe vivax infection

**Figure 3:**
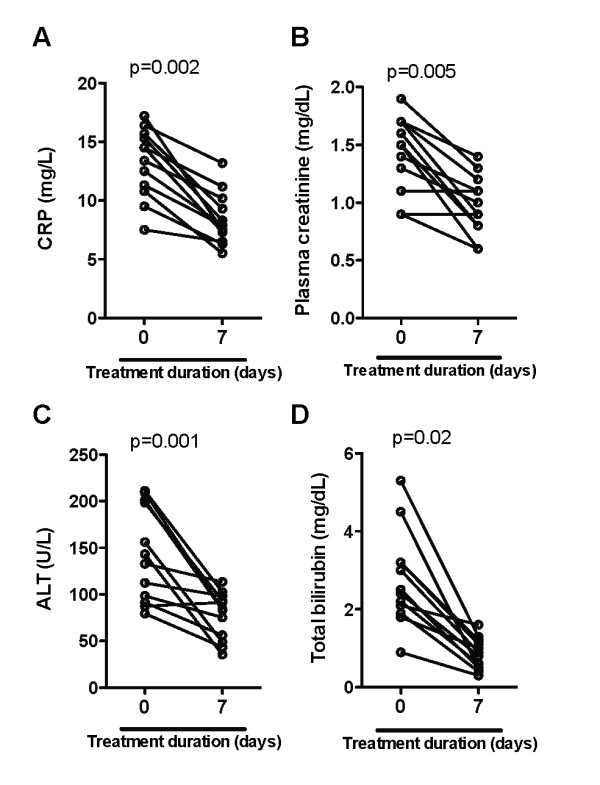
**Kinetic of organ damage indicators during antimalarial treatment in individuals with severe vivax disease**. Plasma levels of (A) CRP, (B) creatinine, (C), ALT and (D) total bilirubin were estimated before treatment (at admission to the Hospital) and after seven days of inhospital care in individuals with severe vivax infection who achieved cure (n = 13). Wilcoxon matched pairs test was performed to calculate the statistical significance. P values are plotted in each graph.

**Figure 4:**
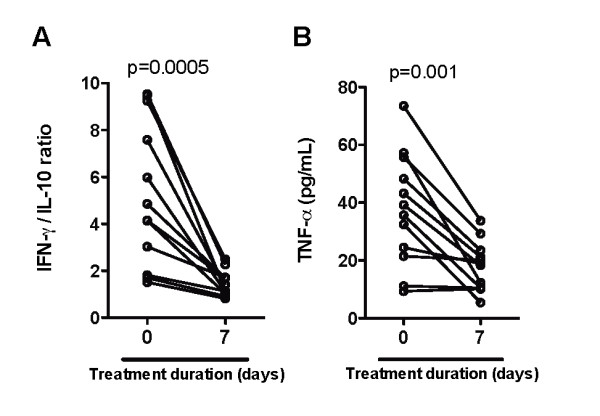
**Kinetic of immunologic indicators during antimalarial treatment in individuals with severe vivax disease**. (A) IFN-gamma/IL-10 ratios and (B) TNF plasma levels were estimated before treatment (at admission to the Hospital) and after seven days of in-hospital care in individuals with severe vivax infection who achieved cure (n = 13). Wilcoxon matched pairs test was performed to calculate the statistical significance. P values are plotted in each graph.

In thirteen patients, who clinically recovered out of nineteen with severe vivax infection, there was an important reduction in the levels of all laboratory parameters of organ damage screened, including plasma CRP (P = 0.002; Figure 3A), creatinine (P = 0.005; Figure 3B), ALT (P = 0.001; Figure 3C) and total bilirubin (P = 0.016; Figure 3D) during anti-parasite treatment. This observation suggests that clinical recovery resulted from a reduction in systemic inflammatory aggression. Regarding the immune markers of pro-inflammatory responses, an important decrease in both IFN-gamma/IL-10 ratios (P = 0.0005; Figure 4A) and TNF levels (P = 0.001; Figure 4B) was noticed during anti-malarial treatment.

## Conclusions

These investigations suggest that different clinical presentations of vivax malaria infection are strongly associated with a potent activation of pro-inflammatory responses and cytokine imbalance. These results are of utmost importance to improve current knowledge about physiopathological concepts of this serious, widespread disease.

## Competing interests

The authors declare that they have no competing interests.

## Authors' contributions

Wrote the paper: BBA and ARF; Performed the field study and clinical examinations: BBA, SMSN and LMAC; Performed the laboratory experiments and data analysis: BBA and JC; Participated in the design of the study and helped with the manuscript: LMAC and AB; Coordinated the study helped to draft the manuscript: MBN. All authors have read and approved the final version of the manuscript.
